# Synergistic interventions to control COVID-19: Mass testing and isolation mitigates reliance on distancing

**DOI:** 10.1371/journal.pcbi.1009518

**Published:** 2021-10-28

**Authors:** Emily Howerton, Matthew J. Ferrari, Ottar N. Bjørnstad, Tiffany L. Bogich, Rebecca K. Borchering, Chris P. Jewell, James D. Nichols, William J. M. Probert, Michael C. Runge, Michael J. Tildesley, Cécile Viboud, Katriona Shea

**Affiliations:** 1 Center for Infectious Disease Dynamics, The Pennsylvania State University, University Park, Pennsylvania, United States of America; 2 Department of Biology, The Pennsylvania State University, University Park, Pennsylvania, United States of America; 3 Lancaster Medical School, Lancaster University, Lancaster, United Kingdom; 4 U.S. Geological Survey, Eastern Ecological Science Center at the Patuxent Research Refuge, Laurel, Maryland, United States of America; 5 Big Data Institute, Li Ka Shing Centre for Health Information and Discovery, University of Oxford, Oxford, United Kingdom; 6 Zeeman Institute for Systems Biology and Infectious Disease Epidemiology Research (SBIDER), Mathematics Institute and School of Life Sciences, University of Warwick, Coventry, United Kingdom; 7 Fogarty International Center, National Institutes of Health, Bethesda, Maryland, United States of America; The University of Melbourne Melbourne School of Psychological Sciences, AUSTRALIA

## Abstract

Stay-at-home orders and shutdowns of non-essential businesses are powerful, but socially costly, tools to control the pandemic spread of SARS-CoV-2. Mass testing strategies, which rely on widely administered frequent and rapid diagnostics to identify and isolate infected individuals, could be a potentially less disruptive management strategy, particularly where vaccine access is limited. In this paper, we assess the extent to which mass testing and isolation strategies can reduce reliance on socially costly non-pharmaceutical interventions, such as distancing and shutdowns. We develop a multi-compartmental model of SARS-CoV-2 transmission incorporating both preventative non-pharmaceutical interventions (NPIs) and testing and isolation to evaluate their combined effect on public health outcomes. Our model is designed to be a policy-guiding tool that captures important realities of the testing system, including constraints on test administration and non-random testing allocation. We show how strategic changes in the characteristics of the testing system, including test administration, test delays, and test sensitivity, can reduce reliance on preventative NPIs without compromising public health outcomes in the future. The lowest NPI levels are possible only when many tests are administered and test delays are short, given limited immunity in the population. Reducing reliance on NPIs is highly dependent on the ability of a testing program to identify and isolate unreported, asymptomatic infections. Changes in NPIs, including the intensity of lockdowns and stay at home orders, should be coordinated with increases in testing to ensure epidemic control; otherwise small additional lifting of these NPIs can lead to dramatic increases in infections, hospitalizations and deaths. Importantly, our results can be used to guide ramp-up of testing capacity in outbreak settings, allow for the flexible design of combined interventions based on social context, and inform future cost-benefit analyses to identify efficient pandemic management strategies.

## Author summary

The global spread of SARS-CoV-2 and the strategies used to manage it have come at significant societal costs. We analyze how mixed control strategies, which utilize interventions that prevent new infections from occurring (e.g., distancing or shut-downs) and others that actively search for and isolate existing infections (here, mass testing), can achieve improved public health outcomes while avoiding severe socio-economic burdens. Our results suggest that increasing testing capacity, including the number of tests available and the speed at which test results are provided, can reduce reliance on costly preventative interventions. Such reduction is possible with more isolation of active infections, including those without reported symptoms. However, failing to maintain preventative interventions without sufficient testing capacity can lead to large increases in infection burdens. By defining the combined effect of these interventions through mathematical models, this study provides insight into relaxation of distancing measures, and lays the groundwork for future public health economics analyses on the cost-effectiveness of combined management strategies.

## Introduction

A wide range of public health interventions have been employed globally in response to the rapid pandemic spread of the SARS-CoV-2 virus, and the associated global burden of COVID-19 disease. In the early days of the pandemic, when little was known about the biology of the virus and the strategies that would most effectively mitigate its transmission [1], many countries implemented strict lockdowns to limit contacts between individuals [2–9]. Though effective in reducing virus spread and minimizing public health burden [7,8,10], significant social and economic costs [11,12] meant these lockdowns have been increasingly unsustainable over time.

Some countries and states are turning to testing and isolation-based strategies as a viable mechanism for control while waiting for effective vaccines and pharmaceuticals to become widely available [13]. The utility of “test, trace, and isolate” strategies has been demonstrated in prior outbreaks of novel infectious diseases, including SARS [14] and Ebola [15], as well as in a multitude of mathematical models [16]. For SARS-CoV-2 specifically, contact tracing and isolation have been shown effective for suppressing early spread, both empirically [17,18] and via modeling [19,20]. However, contact tracing systems have struggled to keep up as caseloads become large [21].

Developments in testing technology and capacity (e.g., United States Food and Drug Administration [22]) have made it potentially feasible and cost-effective to implement largescale testing and isolation strategies for the management of SARS-CoV-2. An increasing body of literature describes the characteristics of mass, population-level testing and isolation programs that successfully control community transmission. These studies emphasize the necessity of testing frequently and providing results quickly, even if this requires sacrifices to test sensitivity (i.e., the ability of the test to correctly identify infected individuals) [23,24]. Unlike symptomatic, or reactive, testing currently being used in many countries, these mass testing strategies require frequently testing a significant proportion of the population, including groups of individuals that may generally not present symptoms (e.g., testing on college campuses [25,26]).

However, testing capacity, though growing, remains relatively low across much of the world [27]. Consequently, few locales are currently in a position to provide large-scale testing as a sole, or even primary, method for SARS-CoV-2 control. In these cases, where the virus cannot be sufficiently contained by testing and isolation and sufficient vaccination thresholds have not been achieved, preventative non-pharmaceutical interventions (NPIs), such as masking or distancing, are necessary to further mitigate viral transmission. Yet, the interaction between preventative NPIs and isolation of active infections, especially mass testing strategies, is even less understood than the effects of either intervention alone (though see McCombs and Kadelka [28] for a COVID-19 example). Identifying effective combination interventions will be crucial as countries move from control strategies based primarily on preventative NPIs towards those that rely more heavily on testing and isolation, and ultimately wide-spread pharmaceutical reduction in burden of disease.

Using multiple interventions has proven effective for controlling transmission in a variety of pathogens (e.g., influenza [29,30], early SARS-CoV-2 in China [18], HIV [31]). However, combining NPIs with testing and isolation is likely to produce complex transmission dynamics, making it difficult to quantify the effects of mixed control. These complex dynamics may arise in part because of differences in the timing of non-pharmaceutical interventions and isolation relative to the infection process. NPIs, like physical distancing, prevent new infections by preemptively reducing contacts across the entire population, whereas isolation of infected individuals reduces subsequent transmission after, but not before isolation. Further, variation in compliance or existing immunity may make the needs of each locale different.

To clarify these decision-critical issues, we develop a mathematical model to explore the interaction between various preventative NPIs and testing and isolation strategies. Our analysis addresses how the testing strategy’s characteristics affect the intensity of the minimally sufficient NPIs to achieve desired public health outcomes, including daily test administration (i.e., how many tests are performed each day), test delays (i.e., the average time from test administration to isolation), and test sensitivity (defined as the accuracy of a test in identifying infectious individuals). By describing the effectiveness of these combined public health interventions, our framework can help, in a time of extreme social, economic, and psychological hardship, to clarify a path to lifting costly NPIs and ramping-up testing capacity, all while maintaining control of transmission.

## Methods

### Model description

**Fig 1:**
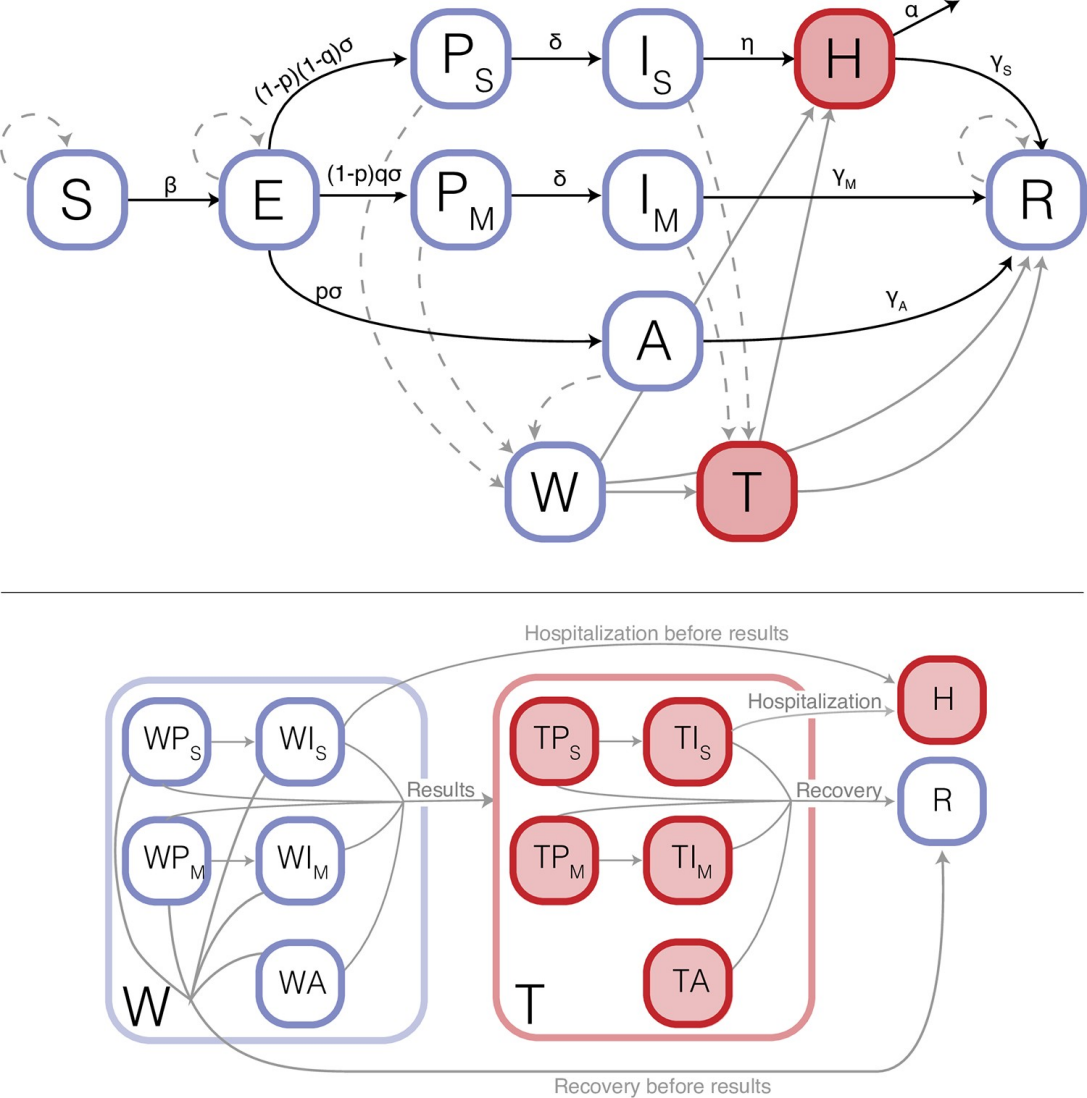
Compartmental structure of the model. Individuals are classified by epidemiological and testing state (S: susceptible, E: exposed, P_S_ and P_M_: presymptomatic transmission for severe and mild infections, respectively, I_S_ and I_M_: symptom reporting for severe and mild infections, respectively, A: asymptomatic infection, H: hospitalized, W: awaiting test results, T: isolated, R: recovered). Pathogen transmission occurs between non-isolated, infected individuals (P, I, A, and W classes) and susceptible individuals (S class). Red filled compartments are isolated, and thus are assumed to not contribute to onward transmission of the pathogen. Solid lines show epidemiological transitions, with parameters to define the rate of transition (see S1 Appendix for full list of transition rates and Table 1 for parametric assumptions). Dashed lines show transitions made through testing, and grey lines show transitions out of waiting and testing classes. The W and T classes are a set of compartments broken down by infection status of tested and isolated individuals respectively (expanded in bottom half of figure). Individuals that report symptoms (I classes) isolate upon test administration, whereas randomly tested individuals await results in the W classes, where they can (1) develop and report symptoms (i.e., move from W_P_ to W_I_), (2) recover or become hospitalized before they receive test results, or (3) receive test results and isolate. Individuals remain isolated until recovery.

Transmission during the acute phase of the SARS-CoV-2 pandemic can plausibly be assumed to follow an SEIR-like epidemiological model (Susceptible-Exposed-Infected-Recovered) because waning of immunity is unlikely within the short (< 6 months) timeframe of our model (Fig 1). We account for key aspects of SARS-CoV-2 biology, including transmission without symptoms (both infections that never develop reportable symptoms and those that are infectious before symptom onset and subsequent reporting) [32,33].

In our model, individuals move from being susceptible to exposed once they have been infected (the E class: infected but not yet infectious). On becoming infectious, individuals move into one of three categories: asymptomatic (i.e., never develop reportable symptoms), mild symptomatic (i.e., develop reportable symptoms that are not severe enough to require hospitalization), or severe symptomatic (i.e., develop reportable symptoms that eventually lead to hospitalization). We distinguish infections based on *reported* symptom status because self-reporting is a primary mechanism for test administration in many countries. We assume that 60% of all infections would eventually develop reportable symptoms [34], and 5% of those with symptoms would be severe enough to require hospitalization [35].

We assume infected individuals who will eventually be symptomatic can start infecting others before symptom onset and reporting. All asymtomatic and mildly symptomatic infections are assumed to eventually recover. Severe symptomatic infections are assumed to be hospitalized and isolated from the population until recovery or death. We assume that recovery confers immunity over the short timeframe of our model in alignment with evidence that SARS-CoV-2 reinfection is rare in the six months following infection [36,37]. We maintain standard assumptions about homogenous mixing, as other modeling studies assessing testing programs have shown little difference between such a model and one with a more realistic contact structure [24]. For a deterministic representation of our model structure, which was based on the model of Davies et al. [38], see S2 Appendix.

### Interventions

We model public health interventions that combine preventative NPIs (e.g., masking, distancing, and lockdown) with testing and isolation. As preventative NPIs reduce the chance of new infection, they are modeled to decrease transmission by some fraction, *d*, which we call the ‘NPI intensity’. Isolation reduces the effective number of infected individuals in the population and thus prevents an infected individual from contributing to onwards transmission.

We assume that testing is the primary mechanism for identifying infections to be isolated. The testing system in our model is characterized by three components: test administration, test delays, and test sensitivity. We explicitly model the number of tests that are administered each day (in some places this may be limited by availability of tests and in others by demand for tests). Testing delays represent the average time, after test administration, it takes to receive test results and subsequently isolate those with a positive diagnosis. We define effective test sensitivity as the percent of actively infectious individuals correctly identified by the diagnostic test. This definition differs from the way sensitivity is determined for other tests (e.g., sensitivity as defined for reverse transcription polymerase chain reaction (RT-PCR) which detects the presence of a viral sequence), but reflects our focus on public health rather than individual patient objectives [23,39,40]. Consistent with our definition of effective sensitivity, we assume that exposed individuals (E class) cannot be detected.

Test allocation follows a consistent state-dependent rule and is executed stochastically using the following sequential steps:

1. Allocate one test for each newly hospitalized patient.
2. Using the remaining tests, allocate one test for each individual that has reported symptoms. The group of individuals reporting symptoms each day includes a constant background rate of non-SARS-CoV-2-infected individuals that report for testing. If there are more individuals reporting symptoms than available tests, allocate tests randomly among the group reporting symptoms.
3. Using the remaining tests, randomly sample individuals without symptoms for testing.
  Whilst we do not claim this is the most efficient way to utilize a fixed number of tests, we believe it represents a simple and logical prioritization that could be implemented. Silverman et al. [41] showed that increases in all-cause care seeking for respiratory infections was highly correlated with cases of COVID-19 early in the pandemic, so we model a constant background rate of symptomatic non-SARS-CoV-2-infected care seeking (test allocation step 2). We further assume that reporting for influenza-like illness (ILI) and SARS-CoV-2 are related, where increases in SARS-CoV-2 reporting also yield higher background care seeking rates. As such, in our model, 0.1(1-*p*)% of the population reports for testing each day despite not being infected with SARS-CoV-2, where *p* is the proportion of SARS-CoV-2 infections that are not reported (Table 1) and 0.1 aligns the order of magnitude with estimates of influenza related medical visits in the United States [42,43]. This simplifying assumption ignores seasonality and potential feedbacks between SARS-CoV-2 interventions and ILI care seeking or prevalence.

**Table 1:** Model parameters. Where no reference is provided, values were assumed. Values shown with an asterisk (*) were considered in sensitivity analyses. The transmission rate, β, was calibrated to yield R_0_ = 2.5 using the next generation matrix method. Transmission rate shown is for primary parameters but was recalibrated for each set of sensitivity analyses.

Biological Parameters	Value
Transmission rate for infected individuals, β	0.502
Relative transmissibility of asymptomatic infections, ρ	1, 0.5* [44]
Time from exposure to infectiousness (days), $\frac{1}{\sigma }$	3 [38]
Time from infectiousness to symptom reporting (days), $\frac{1}{\delta }$	2.1 [38]
Recovery time for asymptomatic infections (days), $\frac{1}{\gamma_{A}}$	5 [38]
Recovery time for mild symptomatic infections (days), $\frac{1}{\gamma_{M}}$	2.9 [38,45,46]
Time from symptom reporting to hospitalization (days), $\frac{1}{\eta }$	2.1 [47]
Recovery time for hospitalized infections (days), $\frac{1}{\gamma_{S}}$	12 [48]
Time from hospitalization to death (days), $\frac{1}{\alpha }$	7.5 [48]
Proportion of infections that are not reported (asymptomatic), *p*	0.4 [34], 0.2*, 0.6*[49]
Proportion of reported (symptomatic) infections requiring hospitalization, *q*	0.05 [35]
**Intervention Parameters**	**Values assessed**
Non-pharmaceutical intervention (NPI) intensity, (1-*d*)	0% to 60%, in 5% increments
Delay between test administration and results, τ	1 hour, and 12 hours to 8 days, in 12-hour increments
Number of tested individuals from class *I*, ω_i_, on a given day, dependent on:	
Test administration	1%, 2.5%, and 5% to 50% in 5% increments
Effective test sensitivity	90%, 100%
Proportion of population without SARS-CoV-2 infection reporting for testing each day	0.001 (1-*p*)

Individuals who report with symptoms are assumed to quarantine immediately upon test administration (test allocation step 2), and those who are randomly sampled isolate only after receiving results (test allocation step 3). We compared this policy with a second, where all individuals isolate only after receiving positive results. All individuals awaiting results are assumed to continue mixing in the population, and thus can transmit virus to others. Note, for simplicity, we omitted isolation of individuals who receive a false positive result, though we acknowledge that imperfect specificity could indirectly affect disease dynamics by impacting long-term compliance with mass testing strategies [50] or further decreasing transmission through additional contact reduction.

### Model implementation, parameterization, and initiation

We implemented the model stochastically using the chain-binomial framework [51–53]. This method assumes that all individuals in the population are classified into a set of compartments (defined for our model in Fig 1), and that individuals move between compartments in discrete time steps. The number of individuals that transition from compartment *i* to compartment *j* at time *t* is determined by a random draw from a binomial distribution, Binomial(t_N_,t_p_), where t_N_ is the number of individuals in compartment *i* at time *t* and t_p_ is the probability of transitioning from *i* to *j*. This probability, t_p_, is derived from the transition rate, r_i,j_(t), between compartments *i* and *j* (defined for our model in Table 1). Given that a transition event happens at rate r_i,j_(t), the number of transitions that occur in a time interval Δt is distributed as Poisson(r_i,j_(t)Δt). Consequently, the probability of no transition events occurring is exp(-r_i,j_(t)Δt) and the probability of transitioning is 1- exp(-r_i,j_(t)Δt). Since we implement our model in daily time steps (i.e., Δt = 1), the transition probability can be simplified to 1- exp(-r_i,j_(t)). S1 Appendix includes a full list of transition probabilities for our model. To avoid negative numbers of individuals, we update the transition probabilities and the resulting number of individuals in each compartment asynchronously in the following order: (1) biological transitions, including infection events, symptom reporting, hospitalization, recovery, and death; (2) test administration and quarantine, if applicable; (3) test results and corresponding isolations for those that are positive.

Model parameters were chosen from literature sources (Table 1). We used the next-generation matrix method [54] to calibrate the transmission parameter to yield an initial, uncontrolled basic reproduction number, R_0_, of 2.5, as estimates of R_0_ before interventions are reported between 2.2 and 2.68 [44,45,55,56] (though some estimates are higher, see [57] for a review). For our analyses, we considered a well-mixed population of 10,000 individuals with low levels of existing immunity in the midst of a growing outbreak of SARS-CoV-2. To generate initial conditions for this scenario, we modeled an initial importation of one mild, presymptomatic infection and one asymptomatic infection that seeded an outbreak which grew unbounded for 7 days (unbounded R_0_ = 2.5) and then experienced moderate growth for 90 days (R_0_ = 1.125 during this period). This setup is not meant to reflect an actual location. We simulated this scenario 1000 times, and the simulation with the 50^th^ percentile of prior immunity (i.e. recovered individuals) on Day 97 was used as our initial conditions (S1 Fig and S1 Table). This approach yielded a population in which approximately 5% of individuals have immunity.

Using these initial conditions, we implemented a range of combination interventions (Table 1) for 30 days, to replicate a short-term planning scenario. Each combination intervention included NPI intensity, test administration, test delay, and effective test sensitivity. NPI intensity ranged from no use of NPIs (0%) to strong reliance on NPIs (60%), a range that captures realistic decreases in transmission due to NPIs (e.g., Flaxman et al. [7]). We modeled test administration from testing 0% to 50% of the population each day. Though this range includes values for daily test administration that have yet to be achieved and sustained, we intentionally include scenarios where tests are widely available to explore the effects of mass testing on transmission. This aligns with other analyses in the literature (e.g., Larremore et al. [24]). To capture an extreme range of testing delays observed during the pandemic, we considered rapid tests (which yield results in an hour, on average) to those that take up to 8 days on average to return results. Lastly, we used 90% and 100% effective test sensitivities. These values are not intended to reflect actual diagnostics, but rather explore the effects of decreases in test sensitivity that may be necessary to achieve large numbers of tests and/or rapid results as it is difficult to measure effective test sensitivity empirically (though see [58,59] for estimates).

We performed 5000 simulations for each potential combination intervention and tracked infection events (i.e., “true” cumulative incidence, regardless of reporting), hospitalizations and deaths. We interpolated the resulting administration-delay-sensitivity-NPI-infection surface to derive contours for 250, 500, and 1000 total infections over 30 days after the intervention change. We ran an additional 5000 simulations for interventions along the 500-infection contour and for interventions where NPI intensity was 5% less than those on the contour. As our model does not consider age or comorbidities, we focus primarily on cumulative infections in our results. We acknowledge that there may be other public health objectives of interest [60,61] and choosing a different objective could alter intervention recommendations [62]. We report all results as median values in alignment with other COVID-19 modeling efforts, including the Forecasting Hub [63] and Scenario Modeling Hub [61].

At the time of model parameterization, there was significant uncertainty about the nature of asymptomatic infections. Additional sensitivity analyses assessed the impact of this uncertainty in two key areas: (1) the proportion of infections that do not develop reportable symptoms (including values that align with more recent reviews [49]) and (2) the relative infectiousness of asymptomatic infections (Table 1). In addition, as immunity levels vary greatly across settings, we quantified the effect of the degree of prior immunity in the population when the intervention change is implemented (S1 Table).

The model and analyses were implemented in R version 3.5.1 [64]. All code is available on the following GitHub repository: https://github.com/eahowerton/COVID19_combined_interventions.

## Results

**Fig 2:**
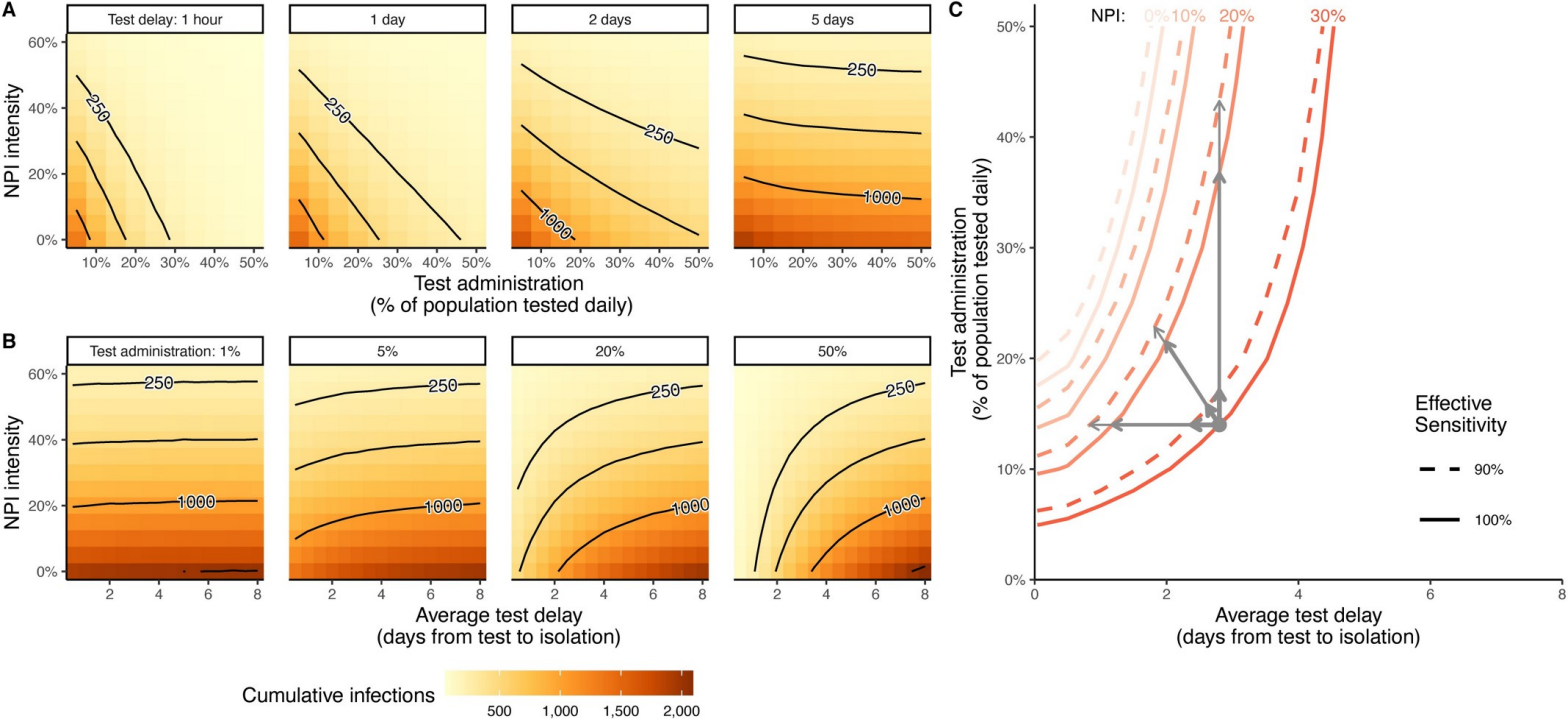
Efficacy of combined interventions including preventative NPIs (e.g., masking, distancing, lockdowns) and testing and isolation. Infections in the 30 days after intervention change (represented as a median of 5000 stochastic simulations) are shown across non-pharmaceutical intervention (NPI) intensities both when test delays are fixed (A) and test administration is fixed (B) for several sample values. In both, isoclines are shown for 250, 500, and 1000 infections, representing potential threshold levels of median infections that a local system can tolerate. Similarly, the test delay and administration required to achieve a given NPI intensity (C) are shown for four potential NPI intensities (line color) across two possible effective test sensitivities (line type; solid = 100%, dashed = 90%), all assuming 500-infection threshold levels (contours for 250- and 1000-infection thresholds shown in S2 Fig). Grey arrows represent sample policy movements between interventions that maintain public health outcomes, where moves can be made by increasing testing administration (vertical arrows), decreasing test delays (horizontal arrows), or a combination (diagonal arrows). Moves can maintain NPI intensity with a less sensitive test (thick arrows), decrease NPI intensity with the same test (medium arrows), and decrease NPI intensity with a less sensitive test (light arrows). See S3 Fig for a version of this figure showing results when individuals who report for testing are assumed to wait for a test result to isolate.

Our analyses describe the combined effect of preventative NPIs and testing and isolation strategies for epidemic control. As expected, high test administration, short testing delays, and more intense NPIs all decrease SARS-CoV-2 infection burdens (Fig 2). Specifically, contours illustrate sets of combined interventions which yield approximately equivalent cumulative infections (median over 5000 simulations; black lines, Fig 2A and 2B). Thus, a public health system willing to tolerate a given level of infection could in principle choose any intervention combination above this contour without exceeding their infection threshold.

For a fixed test delay, testing a larger percent of the population each day can maintain public health outcomes with less reliance on NPIs (Fig 2A). The magnitude of test delays determines the extent to which improvements in test administration permit a reduction in NPIs without increasing disease burden. When delays are relatively long (5 days), testing an additional 5% of the population each day yields at most a 1.5% decrease in the minimally sufficient NPI intensity required to achieve the same level of pandemic control; whereas when tests are rapid, this same increase in test administration yields up to a 12.6% decrease. Since rapid tests isolate infections more quickly and thus have a greater effect on reducing overall transmission, we expect increasing the administration of rapid tests to have the largest impact on the minimally sufficient NPI intensity.

The extent to which minimally sufficient NPIs are decreased by shortening test delays depends on both test administration and current test delays (Fig 2B). When test administration is low (e.g., 1%), which reflects testing levels across much of the world [27], decreasing test delays permits little reduction in NPI intensity. This result is due primarily to the immediate isolation of cases reporting for testing, as in the model most tests are used for this purpose when administration is low. As more tests are administered, the delays associated with these tests have a large influence on the minimally sufficient NPI intensity. In high-administration cases (e.g., 50%), decreases in test delays when delays are already long makes a less substantial difference on NPI intensity, but as test turnaround times become sufficiently fast, small improvements in test delays can yield large changes in the minimally sufficient NPIs. For example, assuming 20% daily test administration and a moderate tolerance threshold (500 infections), a 1-day decrease in testing delays lowers the minimally sufficient NPI intensity by 1.4% when current delays are 7 days, but by 12.8% when current delays are 2 days.

To reduce costs incurred by NPIs or the testing program, policy makers may move between mixed intervention strategies that maintain satisfactory public health outcomes. Fig 2C illustrates how reductions in the intensity of NPIs can be facilitated by improving the performance of a testing program. Consider, for example, a policy that seeks to reduce NPI intensity from 30% to 20% (medium-thickness arrows, Fig 2C) without increasing total infection burden. This can be achieved by reducing test delays by 1.6 days (horizontal arrow), increasing daily testing to an additional 22% of the population per day (vertical arrow), or a smaller combined improvement in both (diagonal arrow). Note that if these gains come at the expense of effective test sensitivity (light-thickness arrows, Fig 2C; e.g., by switching from RT-PCR to rapid diagnostic tests), additional, but small, gains in test turnaround times or administration are necessary (difference between medium and light grey arrows). Similarly, a less sensitive test may offer cost savings; the same reliance on NPIs can be maintained without increasing burdens if the less sensitive test increases administration to at least 3% of the population per day or decreases delays by approximately half a day (heavy-thickness arrows, Fig 2C). As more tests are administered, false negative results accumulate and consequently larger gains in test administration are required to compensate for decreased sensitivity.

Sensitivity analyses show that test administration, test delays, and the minimally sufficient NPI intensity maintain approximately the same contours across uncertainty about prior immunity, percent of infections that are asymptomatic, and the relative infectiousness of asymptomatic infections (S4 Fig). Testing capacity (i.e., administration and delays) that achieves tolerable public health outcomes decreases if there is a higher degree of population immunity, achieved through vaccination for example, and if asymptomatic infections are less infectious than those with symptoms. Changes in the percent of infections that are asymptomatic, which could arise from differences in age structure or access to care, has little effect on the minimally sufficient NPI intensity for a given testing capacity.

**Fig 3:**
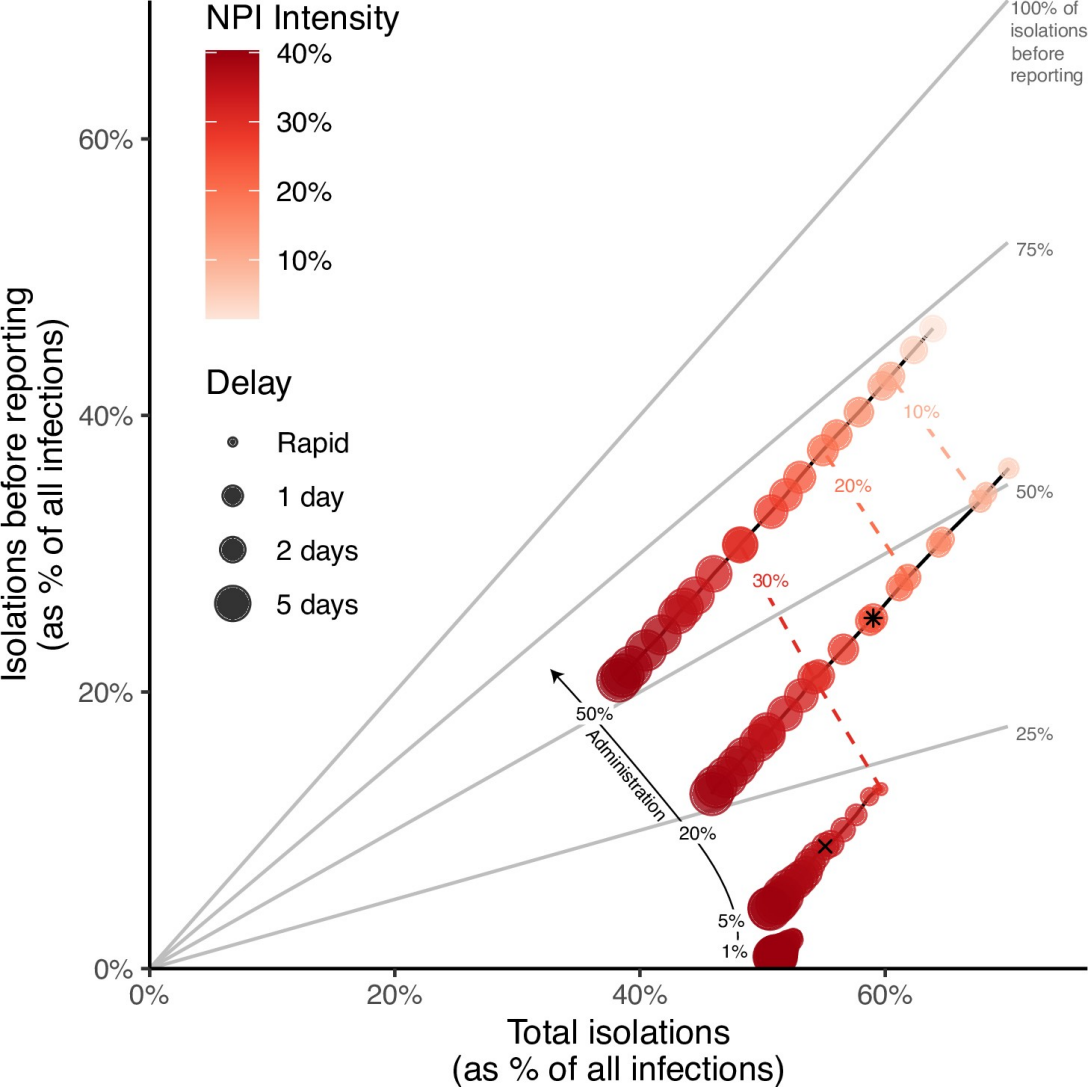
Total isolations, and of those how many were individuals without symptoms (presymptomatic and asymptomatic infections), for various combined interventions. Each dot represents an intervention combination that results in a median of 500 infections over the 30 days after intervention change (i.e., falls along the contour in Fig 2). For each intervention combination, we record the percent of all infections that are isolated while infectious (x-axis) and isolated before reporting (y-axis). Dot color represents non-pharmaceutical intervention (NPI) intensity and dot size represents test delay of the corresponding strategy. Outcomes are shown for a fixed number of test administration levels (1%, 5%, 20%, 50% of the population tested per day), and dashed lines connect strategies with the same NPI intensity across administration levels. Grey lines serve as a reference to show the percent of all isolations that occurred in those without reported symptoms. Points with cross and asterisk are discussed in the text. See S6 Fig for a version of this figure comparing results when individuals with symptomatic infections are assumed to wait for a test result to isolate.

Combined interventions with less intense NPIs can achieve equivalent public health outcomes because they isolate a greater number of infected individuals via testing (Fig 3). The minimally sufficient NPIs are lowest when a significant proportion of infections are isolated before symptom reporting (i.e., presymptomatic and asymptomatic infections), which increases with higher test administration and shorter test delays. For example, consider two interventions with NPI intensity of 35% and 25%. Public health outcomes are maintained under 35% NPI intensity when tests are available for 5% of the population each day and delays are 2 days; this results in isolating 55% of all infections, with 9% of the infections being isolated before symptom reporting (cross, Fig 3). In contrast, reducing NPIs to 25% can maintain public health outcomes when tests are available for 20% of the population if delays are at most 2.5 days. Such an intervention results in a similar percent of infections being isolated (58%) but now 25% of those infections are isolated before reporting symptoms (asterisk, Fig 3). Similarly, isolating reported infections immediately upon test administration has a substantial impact on the minimally sufficient NPI intensity because fewer infected individuals recover before being isolated, especially when test delays are long (S5 Fig).

**Fig 4:**
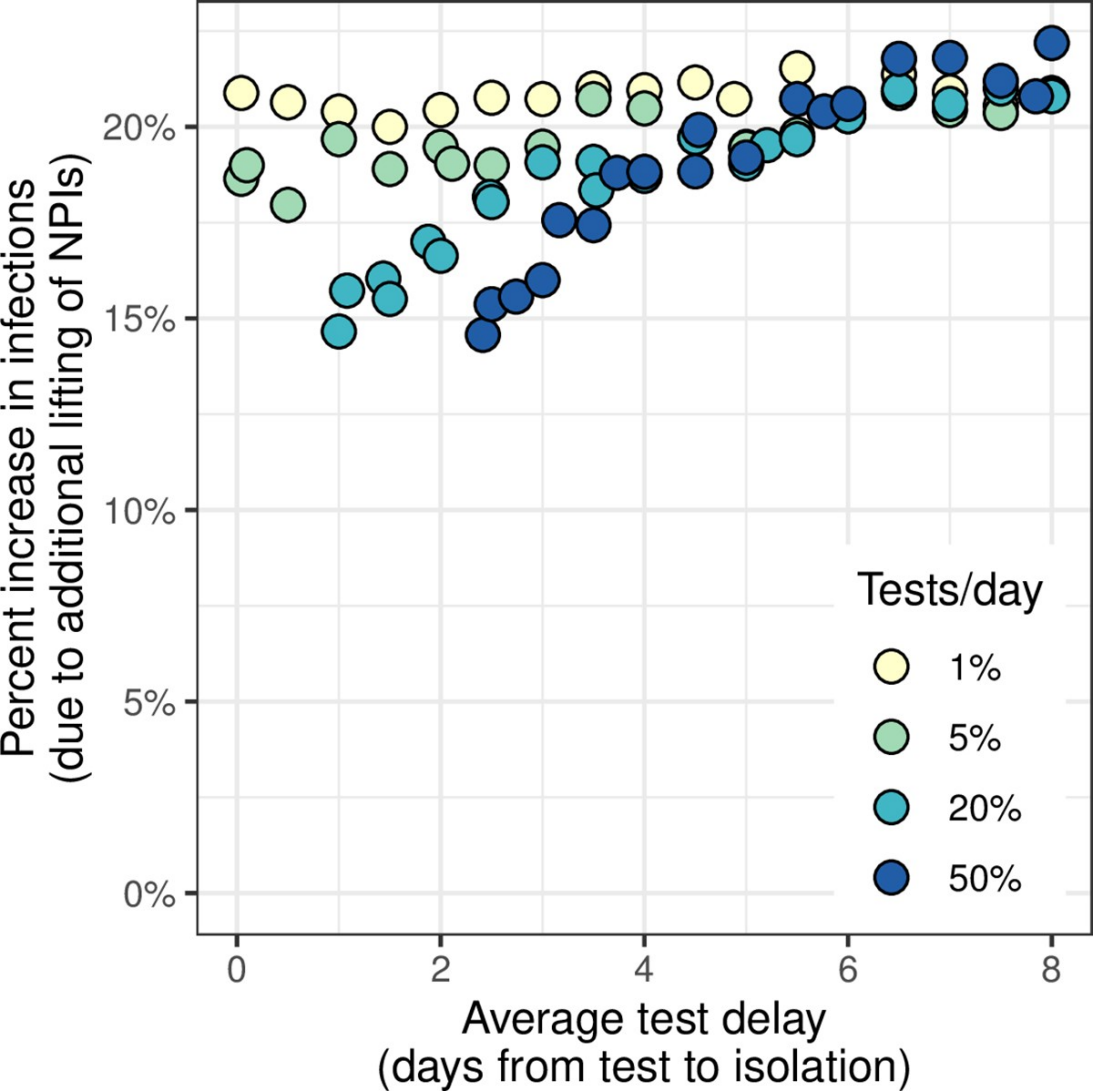
Increases in infections due to additional lifting of NPIs. Increases measure the change in cumulative infections in the 30 days after intervention implementation caused by lifting non-pharmaceutical interventions (NPIs) an additional 5% beyond the recommended NPI intensity. Each point represents a unique combination of test delay (x-axis) and test administration (color). The percent increase was calculated as (O_L_−O_R_)/O_R_, where O_L_ is the number of infections that occur with additional NPI lifting, and O_R_ is the outcome when intervention adheres to NPI recommendations as indicated along the 500-infection contour in Fig 2. Points are not shown for interventions with short delays and high administration because such combinations yield fewer than 500 median cumulative infections without any NPIs (e.g., see Fig 2A: 1 hour, or Fig 2B: 50% administration).

Lifting NPIs an additional 5% (beyond what is recommended along the contours in Fig 2), leads to an average 20% increase in infections over 30 days (Fig 4). Long test delays and low test administration both exacerbate increases caused by additional reopening. For example, assuming 20% test administration, lifting NPIs by an additional 5% yields a 20% increase in infections when delays are 7 days, and only a 14% increase when delays are 1 day. Similarly, total infections increase 21% when 1% of the population is tested daily, compared to 14% when 20% is tested daily, assuming delays are 1 day. Larger increases correspond to intervention strategies that rely more heavily on NPIs for transmission control (i.e., long test delays or low test administration). Note that the appearance of a slightly higher increase in infections under very long test delays and 50% test administration is a result of a large number of individuals passing through both the asymptomatic and waiting classes. Because the model does not track individuals, passing through both classes can yield a minor increase in the infectious period; the effect is negligible for most settings but results in a minimial increase in infections under this most extreme intervention scenario. We note that this slight bias is within the range of stochastic variation.

## Discussion

Large-scale testing and isolation provides promise as a public health intervention that both maintains control of the COVID-19 pandemic and enables continued social and economic activity [13]. In this paper, we discuss how testing and isolation strategies can be combined with preventative non-pharmaceutical interventions such as lockdowns, physical distancing and mask wearing to reduce socio-economic and psychological burdens. Identifying effective combination interventions is instrumental for safely ramping-up testing capacity and lifting costly NPIs without causing adverse public health outcomes. Our analysis demonstrates interactions between multiple interventions focused on this goal. It further provides a decision-making framework with numerous equally effective options for meeting tolerable infection thresholds. Such flexibility is crucial for successfully implementing interventions across a range of social contexts, including situations with different NPI compliance levels.

Maintaining NPIs will be critically important to ensure control of SARS-CoV-2 while continuing to build testing capacity, where capacity refers to the overall ability to process tests including administration and delays. When few tests are available or delays are long, relatively strong NPIs must be continued to keep public health burdens to manageable levels. Importantly, both high testing capacity and short test delays must be achieved in order to safely reduce NPIs to low levels. Failing to maintain sufficient NPIs can lead to considerable increases in infections, hospitalizations and deaths. These increases are most extreme when testing capacity is small since management strategies rely heavily on NPIs in these cases. Higher degrees of immunity in the population, enabled by vaccination for example, reduce testing capacity and NPIs needed to control burdens, though continuing these interventions through vaccine rollout will be critically important to prevent interim increases in cases. Our results emphasize the importance of minimizing time from infection to isolation, as shown in other modeling studies [19,24,28]. Long testing delays significantly limit the range of interventions available to meet public health requirements. As such, the development and use of rapid tests provides decision-makers with more intervention options, including a shift away from burdensome and costly NPIs or an ability to compensate for decreases in compliance.

Swiftly identifying and isolating infections in individuals with symptoms or those reporting for testing can decrease reliance on NPIs; yet, public health outcomes can be maintained under the lowest NPI levels when interventions isolate a large proportion of asymptomatic infections. Increases in test administration enable the testing of individuals who do not self-report and subsequent isolation of asymptomatic infections. However, systems which fail to test individuals without reported symptoms, or those that do not provide access to testing for all, will not achieve the same results no matter how many tests are performed. Thus, we emphasize the importance of building testing systems to screen individuals without symptoms as a necessary step in the process of decreasing our reliance on NPIs, especially when pharmaceutical alternatives are not readily available. Testing in asymptomatic populations can also allow more accurate measurement of epidemiological dynamics and estimation of key parameters, which are crucial for epidemiological modeling for future decision-making [39].

Potential policy options are limited by resource, compliance, and technological constraints. As such, feasible interventions represent only a limited set of the combinations we explore in this paper. Outlining the most efficient ways to move between these possible interventions, including health economic modeling to investigate cost-benefit tradeoffs (e.g., Lee et al. [65]), is an important next step for future research and could build directly on the outputs of our study. For example, some NPIs (e.g., masking and between-individual physical distancing, or “staying six-feet apart”), though less effective in reducing transmission, are much less costly than lockdowns. This difference means it may be more cost effective to continue low-cost preventative interventions with a mass testing strategy, as we show a small degree of preventative intervention has a meaningful effect on required testing capacity. Considering both short- and long-term costs, across many domains including public health, social, and economic, will be important for designing readily achievable and sustainable strategies.

In addition to practical constraints, uncertainty further inhibits decision making. An ability to precisely estimate the effect of an NPI on transmission is a difficult task retrospectively [66,67], and even more challenging prospectively. Our results show the sensitivity of public health outcomes to NPI intensity, and thus an ability to accurately predict and estimate the effect of NPIs on transmission is central to implementing this approach and should be the focus of future research. Advances in behavioral science on differential compliance across interventions and statistical methods for estimating transmission-reducing effects of interventions, given compliance, would allow us to integrate across biological and behavioral uncertainty to identify strategies with the greatest chance of success in a given context. Though some countries (e.g., Slovakia [13], South Korea and Singapore [68]) and institutions (e.g., some university campuses [25]) have achieved remarkably high testing rates, we acknowledge that widely implementing mass testing will require broad buy-in from institutions (e.g., governments and employers) and individuals (i.e., willingness on the part of people to participate in testing and abide by isolation), as well as proper technology, such as low-cost, at-home, rapid diagnostic tests [23,40,69].

Our model framework is intentionally designed to be general in order to allow for exploration of epidemic outcomes under combined interventions. As such, the estimates in this paper are not meant to provide exact testing requirements, but rather to draw general conclusions about the relationship between NPI intensity and testing and isolation strategies for controlling COVID-19. As a result of this generality, there are components of SARS-CoV-2 dynamics that are not included explicitly, such as reintroduction [70], potential reinfection [71], age structure [8,9,38], viral-load dynamics [24,65], time-varying biological and intervention parameters [72], and widespread vaccination, though our framework supports the inclusion of these components. Further, we make specific assumptions about the testing system that warrant additional exploration, including the effect of daily testing (as compared to using the same number of tests at other frequencies) and test allocation. Finally, we make no assumptions about the relative costs of different intervention combinations. A comprehensive cost-benefit analysis would be beneficial to determine how to prioritize different intervention combinations to balance direct public health burden from disease, while recognizing important downstream costs from quarantines, shut-downs, and isolations.

Like other modeling [24,26,28] and empirical [13] studies, our results suggest that rapid or more available tests provide increased control of transmission and can also compensate for decreases in test sensitivity typically associated with rapid tests. However, unlike previous work, we define how these testing characteristics interact with NPIs in a model framework that explicitly accounts for constraints on daily test administration and non-random test allocation. We show that increases in test administration have the greatest impact when test delays are small, less than 48 hours, and decreases in test delays enable the most reopening when delays are already relatively short or more tests are administered. Lifting NPIs too rapidly can lead to significant increases in infections, hospitalizations, and deaths in the 30 days after the intervention change.

Strategically combining public health interventions could allow us to maintain control of SARS-CoV-2, achieve increasing degrees of social and economic activity, and therefore decrease the significant overall societal costs incurred because of the COVID-19 pandemic. Understanding the efficacy of combined public health interventions is a key first step in identifying cost-effective ways to manage the pandemic.

## Supporting information

S1 Fig Simulations used to generate initial conditions. Grey lines show all simulations, and black line shows simulation selected as initial conditions for interventions. See Methods for details on how initial conditions were generated.
(TIF)

S2 Fig Contours for additional tolerance thresholds. Test delay and administration required to achieve a given non-pharmaceutical intervention (NPI) intensity (line color) are shown for two possible test sensitivities (line type; solid = 100% effective test sensitivity, dashed = 90% effective test sensitivity), across 250- and 1000-infection thresholds.
(TIF)

S3 Fig Outcomes when symptomatic infections are isolated upon receiving test results. Infections in the 30 days after intervention change (represented as a median of 5000 stochastic simulations) are shown across non-pharmaceutical intervention (NPI) intensities both when test delays are fixed (A) and test administration is fixed (B) for several sample values. In both, isoclines are shown for 250, 500, 1000, and 2000 infections. Similarly, the test delay and administration required to achieve a given NPI intensity (C) are shown for five potential NPI intensities (line color) across two possible test sensitivities (line type; solid = 100% sensitivity, dashed = 90% sensitivity), all assuming 500-case tolerance. Note: colors have been rescaled compared to main text.
(TIF)

S4 Fig Results of sensitivity analyses. Sensitivity analyses considered the effect of assumptions about (A) prior immunity; (B) the percent of infections that are asymptomatic; and (C) the relative infectiousness of asymptomatic infections. Each panel shows contours representing combination interventions (test delay, x-axis, and test administration, y-axis) that yield equivalent public health outcomes (as in Fig 2C). In all panels, contours show interventions which yield a median 250 infections in the 30 days following intervention change. The base scenario, as reported in the main text and represented with a black line in each panel, has 4.77% prior immunity, 40% asymptomatic infections, and equal infectiousness between symptomatic and asymptomatic infections.
(TIF)

S5 Fig Changes in total isolations and NPI intensity due to immediate quarantine of symptomatic infections. Interventions which quarantine symptomatic infections immediately upon test administration are compared to the corresponding intervention (identical test administration and delay, with non-pharmaceutical intervention (NPI) intensity adjusted to yield 500-infection tolerance threshold, i.e., chosen to fall along the isocline shown in Fig 2) where symptomatic infections wait to be isolated until receiving test results. Differences are shown for four sample test administrations (color) by test delay (dot size). The difference was calculated as O_D_−O_I_, where O_D_ is the outcome (total isolations or minimally sufficient NPI intensity) when symptomatic isolation is delayed based on time to test results, and O_I_ is the outcome under immediate quarantine of symptomatic infections.
(TIF)

S6 Fig **Isolations comparing strategies which isolate symptomatic infections immediately (red) vs. upon receiving results (grey).** See Fig 3 legend for figure description. Note: red points shown are identical to those shown in Fig 3, with colors rescaled to match those in grey, and are provided as a reference.
(TIF)

S1 Appendix Transitions between model compartments. (PDF)

S2 Appendix Deterministic model representation. (PDF)

S1 Table Initial conditions. Initial conditions representing moderate levels of prior immunity at the time of intervention change. All W classes and T classes are represented by W_i_ and T_i_, respectively. Increased levels of prior immunity were also considered in sensitivity analyses (denoted by *).
(PDF)
