# Supplementary figures and images for: Synergistic interventions to control COVID-19: Mass testing and isolation mitigates reliance on distancing
Source: PLoS Comput Biol. 2021 Oct 28;17(10):e1009518. doi: 10.1371/journal.pcbi.1009518 (PMC8553097; doi:10.1371/journal.pcbi.1009518)

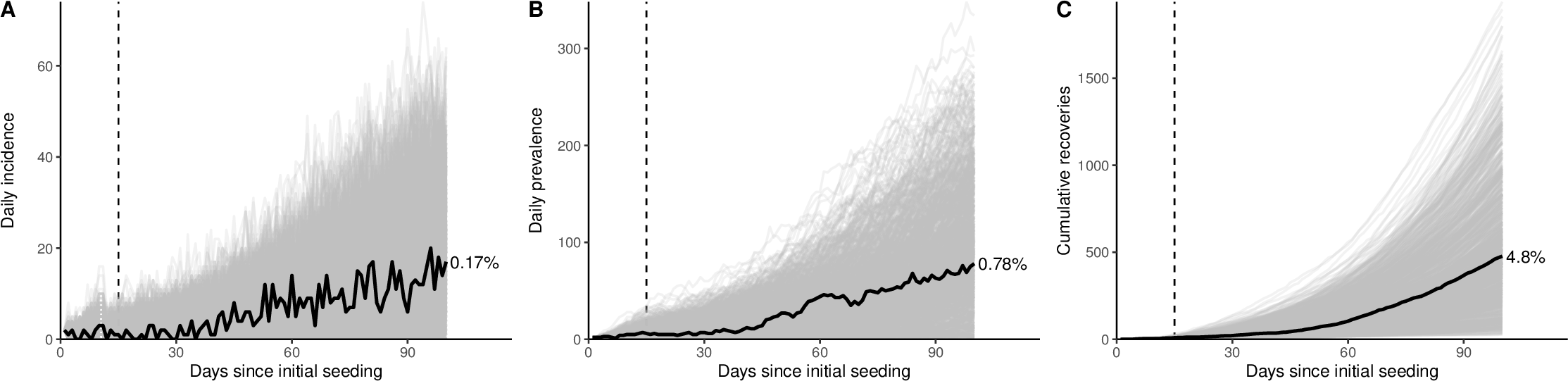

Supplement: S1 Fig — Grey lines show all simulations, and black line shows simulation selected as initial conditions for interventions. See Methods for details on how initial conditions were generated. (TIF) [file pcbi.1009518.s001.tif]

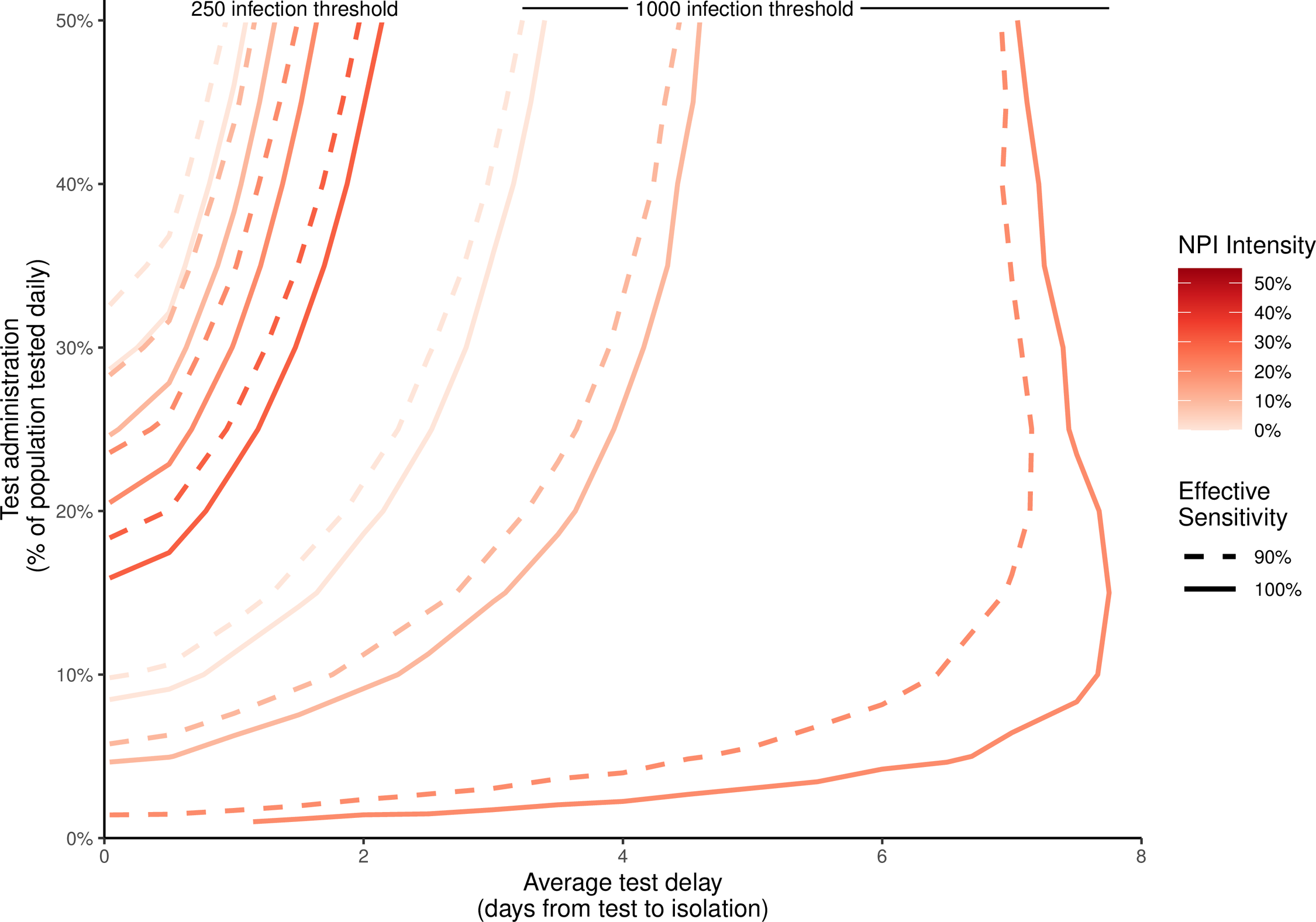

Supplement: S2 Fig — Test delay and administration required to achieve a given non-pharmaceutical intervention (NPI) intensity (line color) are shown for two possible test sensitivities (line type; solid = 100% effective test sensitivity, dashed = 90% effective test sensitivity), across 250- and 1000-infection thresholds. (TIF) [file pcbi.1009518.s002.tif]

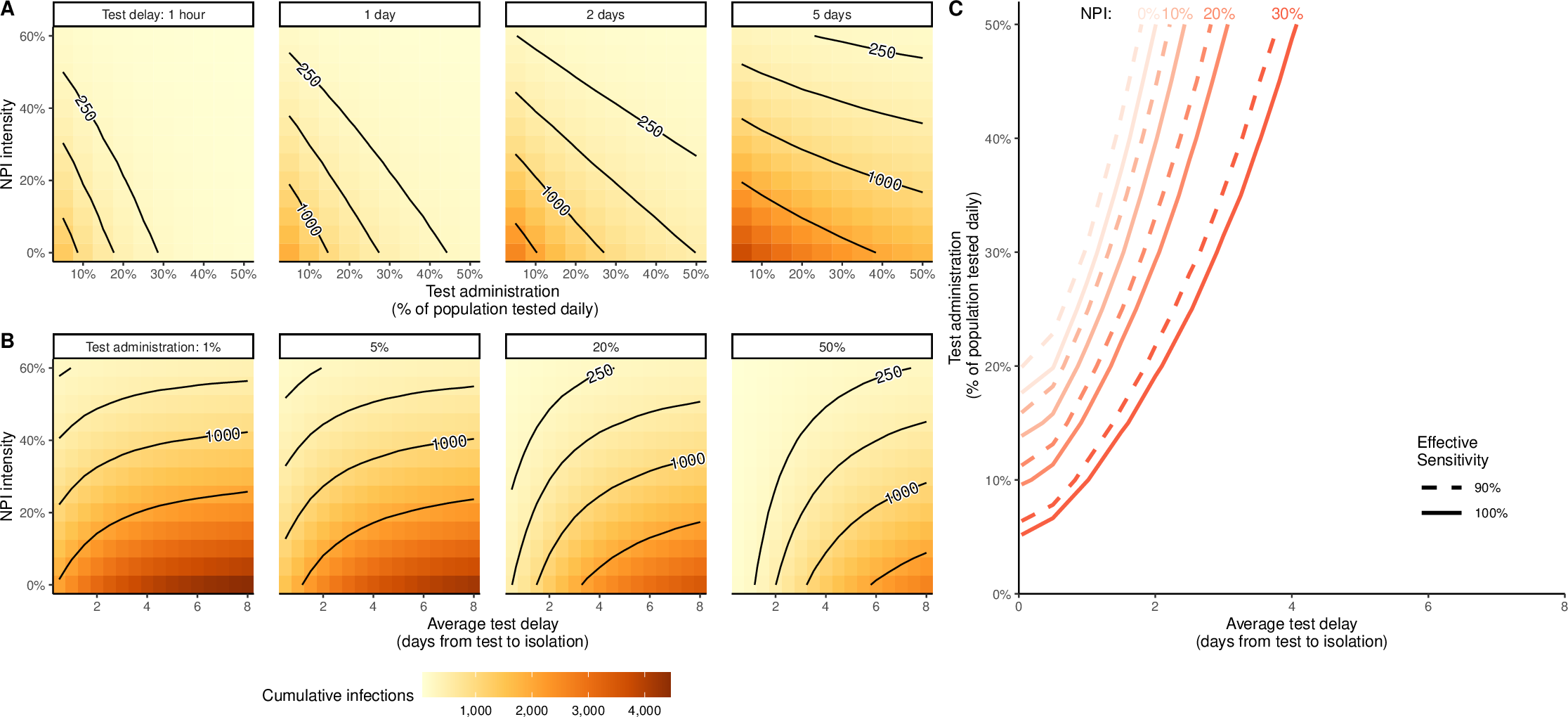

Supplement: S3 Fig — Infections in the 30 days after intervention change (represented as a median of 5000 stochastic simulations) are shown across non-pharmaceutical intervention (NPI) intensities both when test delays are fixed (A) and test administration is fixed (B) for several sample values. In both, isoclines are shown for 250, 500, 1000, and 2000 infections. Similarly, the test delay and administration required to achieve a given NPI intensity (C) are shown for five potential NPI intensities (line color) across two possible test sensitivities (line type; solid = 100% sensitivity, dashed = 90% sensitivity), all assuming 500-case tolerance. Note: colors have been rescaled compared to main text. (TIF) [file pcbi.1009518.s003.tif]

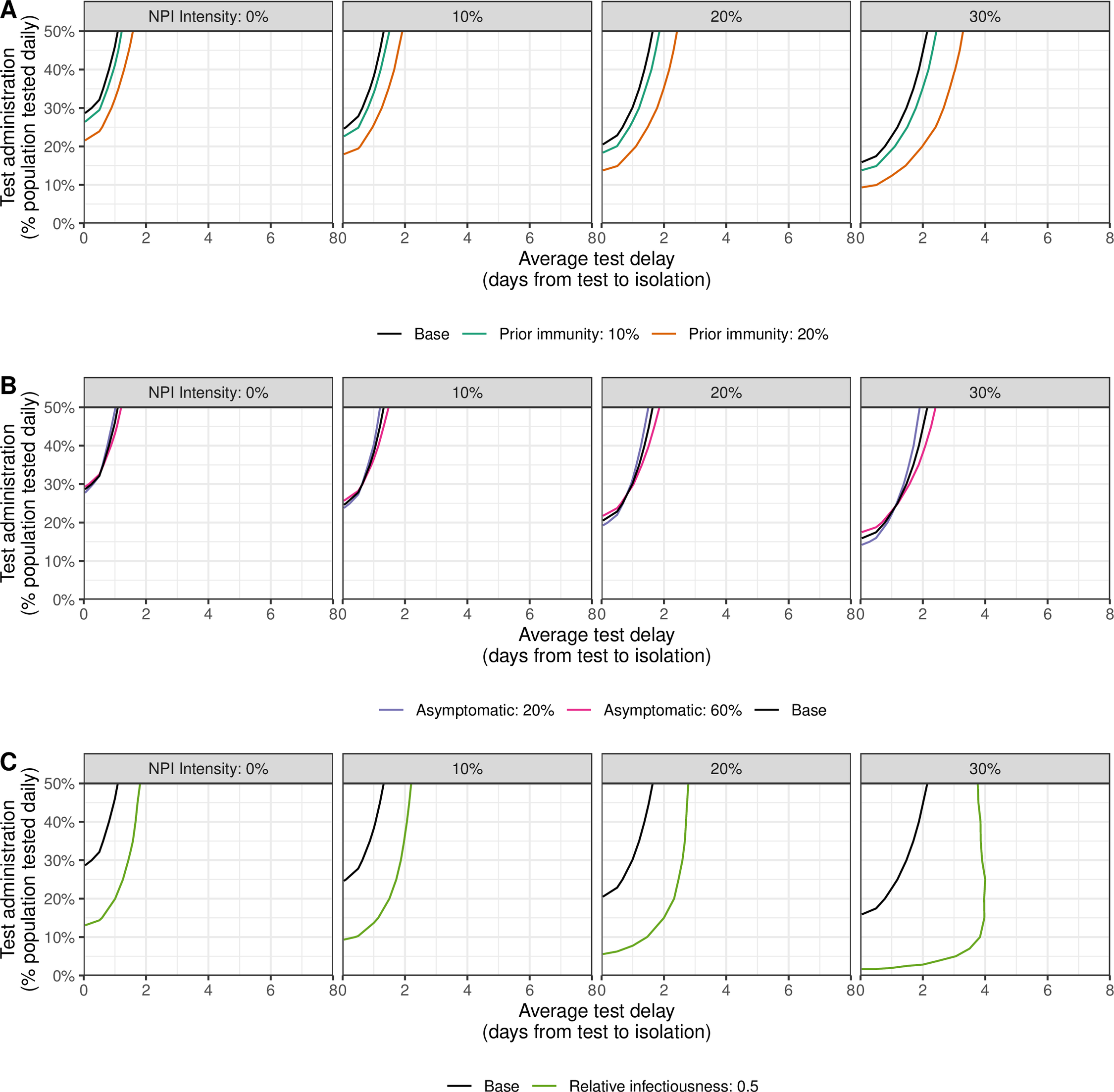

Supplement: S4 Fig — Sensitivity analyses considered the effect of assumptions about (A) prior immunity; (B) the percent of infections that are asymptomatic; and (C) the relative infectiousness of asymptomatic infections. Each panel shows contours representing combination interventions (test delay, x-axis, and test administration, y-axis) that yield equivalent public health outcomes (as in Fig 2C). In all panels, contours show interventions which yield a median 250 infections in the 30 days following intervention change. The base scenario, as reported in the main text and represented with a black line in each panel, has 4.77% prior immunity, 40% asymptomatic infections, and equal infectiousness between symptomatic and asymptomatic infections. (TIF) [file pcbi.1009518.s004.tif]

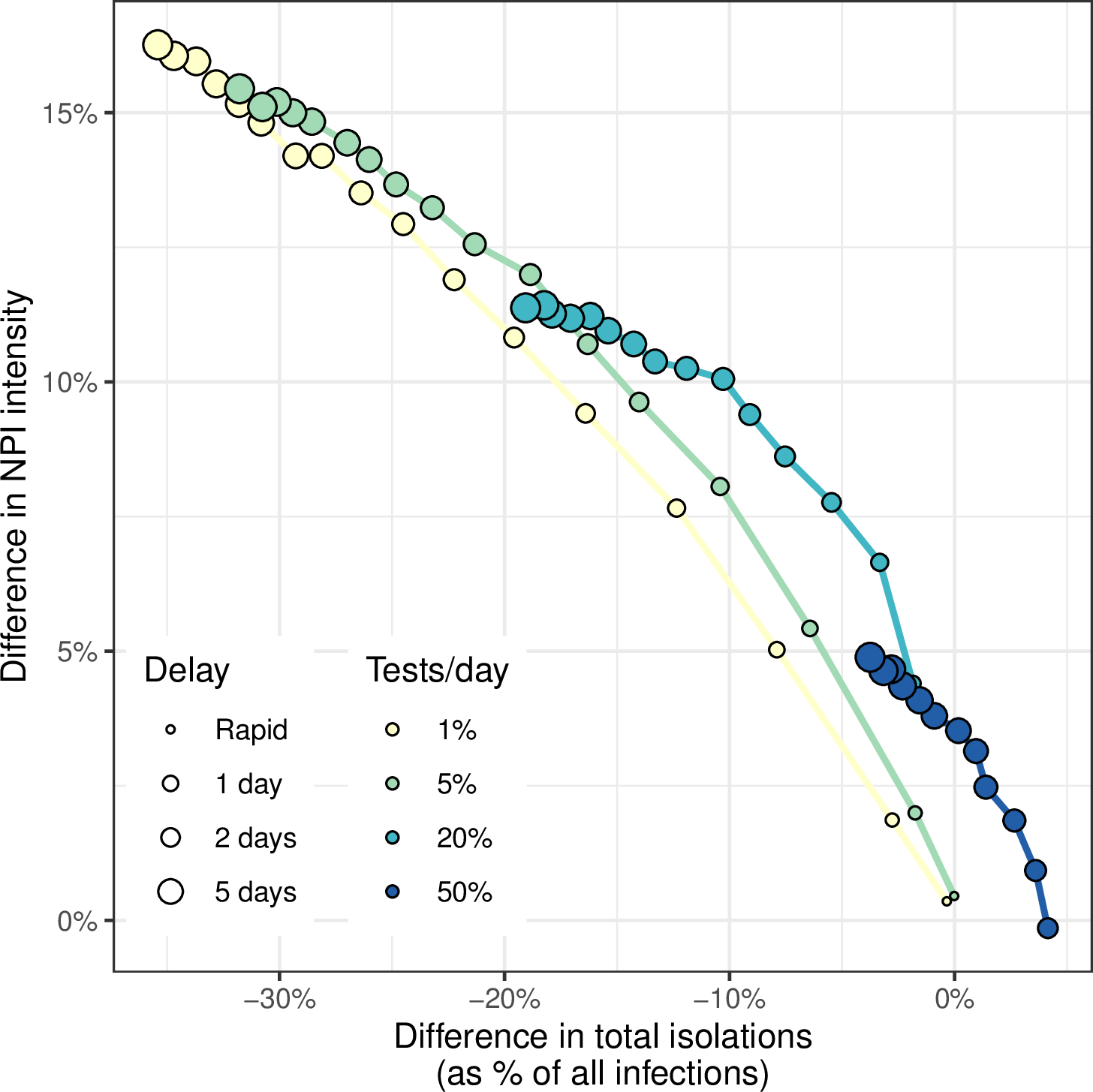

Supplement: S5 Fig — Interventions which quarantine symptomatic infections immediately upon test administration are compared to the corresponding intervention (identical test administration and delay, with non-pharmaceutical intervention (NPI) intensity adjusted to yield 500-infection tolerance threshold, i.e., chosen to fall along the isocline shown in Fig 2) where symptomatic infections wait to be isolated until receiving test results. Differences are shown for four sample test administrations (color) by test delay (dot size). The difference was calculated as OD−OI, where OD is the outcome (total isolations or minimally sufficient NPI intensity) when symptomatic isolation is delayed based on time to test results, and OI is the outcome under immediate quarantine of symptomatic infections. (TIF) [file pcbi.1009518.s005.tif]

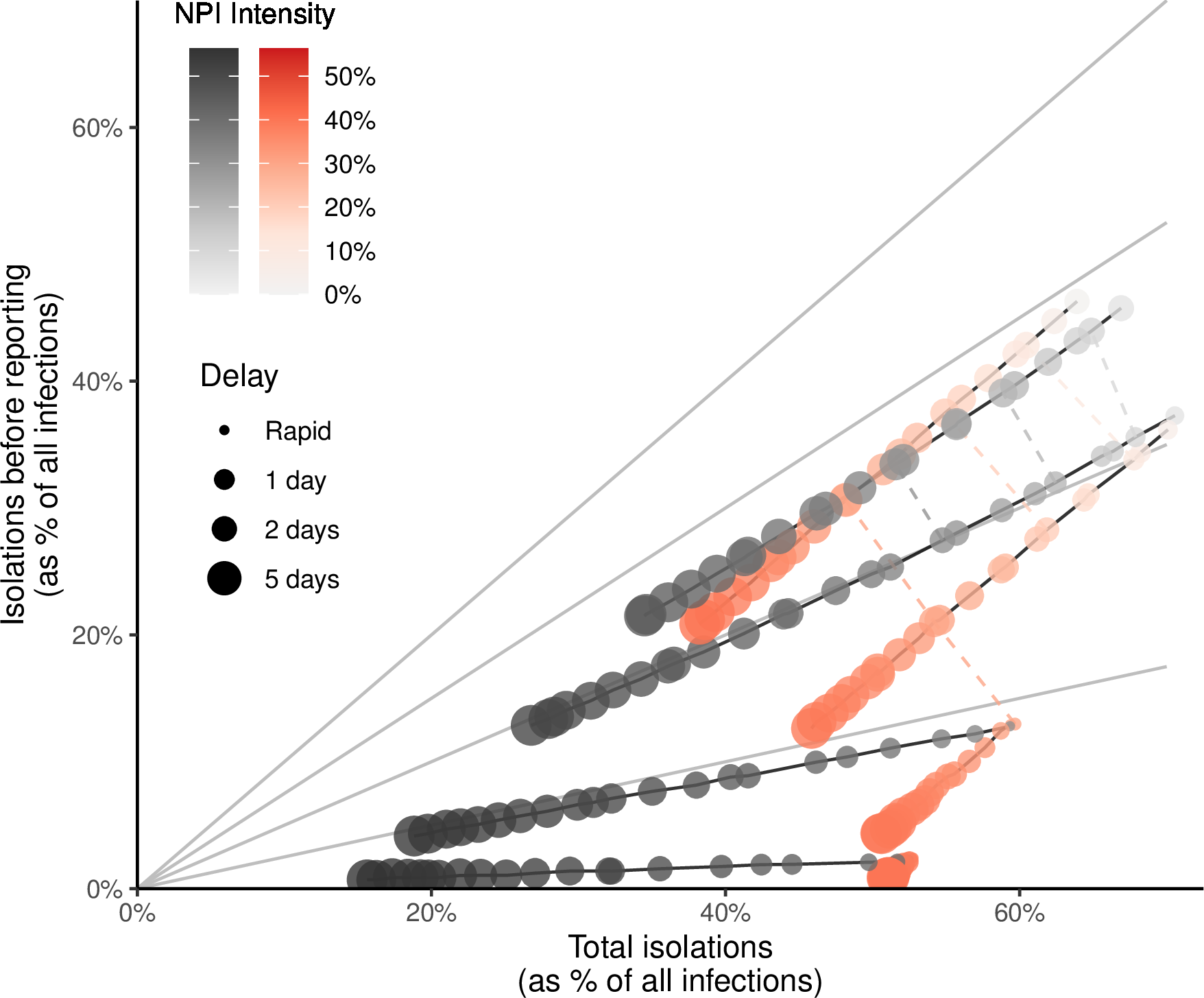

Supplement: S6 Fig — Isolations comparing strategies which isolate symptomatic infections immediately (red) vs. upon receiving results (grey). See Fig 3 legend for figure description. Note: red points shown are identical to those shown in Fig 3, with colors rescaled to match those in grey, and are provided as a reference. (TIF) [file pcbi.1009518.s006.tif]
