# Supplementary material for: Synergistic interventions to control COVID-19: Mass testing and isolation mitigates reliance on distancing
Source: PLoS Comput Biol. 2021 Oct 28;17(10):e1009518. doi: 10.1371/journal.pcbi.1009518 (PMC8553097; doi:10.1371/journal.pcbi.1009518)
Supplement: S1 Appendix — (PDF) [file pcbi.1009518.s007.pdf]

S1 Appendix. Transitions between model compartments.

Following the chain-binomial framework, the number of transition events that occur at each time step is a random draw from Binomial($D_i(t)$, $1 - exp(-r_{i,j}(t))$ where $D_i(t)$ is the number of individual in compartment i at time t and $r_{i,j}(t)$ as defined below. To keep the table concise, we define the transmission rate as $b(t) = \frac{d\beta(I_M(t)+I_S(t)+W_{I_M}(t)+W_{I_S}(t)+\rho(A(t)+P_M(t)+P_S(t)+W_A(t)+W_{P_M}(t)+W_{P_S}(t)))}{N(t)-H(t)-\sum T_k(t)}$.

	State ($i \to j$)	Description	Transition rate, $r_{i,j}(t)$
Before testing	$S \to E$	transmission	b(t)
	$E \to P_M$	become infectious, mild infections	$(1-p)(1-q)\sigma$
	$E \to P_S$	become infectious, severe infections	$(1-p)q\sigma$
	$E \to A$	become infectious, asymptomatic infections	$p\sigma$
	$P_M \to I_M$	develop and report mild symptoms	δ
	$P_S \to I_S$	develop and report severe symptoms	δ
	$A \to R$	asymptomatic recovery	γ_A
	$I_M \to R$	mild symptomatic recovery	γ_M
	$I_S \to H$	hospitalization	η
Administer tests	$P_M \to W_{P_M}$	test presymptomatic infection, await results	See Interventions subsection for details
	$P_S \to W_{P_S}$	test presymptomatic infection, await results	
	$A \to W_A$	test asymptomatic infection, await results	
	$I_M \to W_{I_M}$	test mild reported infection, isolate immediately	
	$I_S \to W_{I_S}$	test severe reported infection, isolate immediately	
Await results	$W_{P_M} \to W_{I_M}$	develop and report mild symptoms	δ
	$W_{P_S} \to W_{I_S}$	develop and report severe symptoms	δ
	$W_A \to R$	asymptomatic recovery before results	γ_A
	$W_{I_M} \to R$	mild symptomatic recovery before results	γ_M
	$W_{I_S} \to H$	hospitalization before results	η
Receive results	$W_{P_M} \to T_{P_M}$	true positive result while presymptomatic, isolate	τ
	$W_{P_S} \to T_{P_S}$	true positive result while presymptomatic, isolate	τ
	$W_A \to T_{P_S}$	true positive result while asymptomatic, isolate	τ
	$W_{I_M} \to T_{I_M}$	true positive result after mild symptoms, isolate	τ
	$W_{I_S} \to T_{I_S}$	true positive result after severe symptoms, isolate	τ
In isolation	$T_{P_M} \to T_{I_M}$	develop and report mild symptoms while isolated	δ
	$T_{P_S} \to T_{I_S}$	develop and report severe symptoms while isolated	δ
	$T_A \to R$	asymptomatic recovery while isolated	γ_A
	$T_{I_M} \to R$	mild symptomatic recovery while isolated	γ_M
	$T_{I_S} \to H$	hospitalization while isolated	η
	$H \to R$	hospitalized recovery	γ_S
	$H \to D$	death	α
