# Supplementary material for: Synergistic interventions to control COVID-19: Mass testing and isolation mitigates reliance on distancing
Source: PLoS Comput Biol. 2021 Oct 28;17(10):e1009518. doi: 10.1371/journal.pcbi.1009518 (PMC8553097; doi:10.1371/journal.pcbi.1009518)
Supplement: S2 Appendix — (PDF) [file pcbi.1009518.s008.pdf]

S2 Appendix. Deterministic model representation.

The deterministic skeleton of our model is defined mathematically by the below set of coupled differential equations. In order to study the cloud of likely stochastic outcomes, we discretized time in daily time steps and implemented the multi-compartmental chain-binomial version of the model.

$$\underset{\text{susceptible}}{\dot{S}} = \underset{\text{transmission, reported symptoms}}{\frac{-d\beta S(I_M + I_S + W_{I_M} + W_{I_S})}{N - H - \sum T_i}} + \underset{\text{transmission, unreported symptoms}}{\frac{-d\rho\beta S(A + P_M + P_S + W_A + W_{P_M} + W_{P_S})}{N - H - \sum T_i}} \tag{1}$$

$$\underset{\text{exposed}}{\dot{E}} = \underset{\text{transmission, reported symptoms}}{\frac{d\beta S(I_M + I_S + W_{I_M} + W_{I_S})}{N - H - \sum T_i}} + \underset{\text{transmission, unreported symptoms}}{\frac{d\rho\beta S(A + P_M + P_S + W_A + W_{P_M} + W_{P_S})}{N - H - \sum T_i}} - \underset{\text{become infectious}}{\sigma E} \tag{2}$$

$$\underset{\substack{\text{presymptomatic}\\ \text{eventually mild}}}{\dot{P_M}} = \underset{\text{become infectious}}{(1-p)(1-q)\sigma E} - \underset{\substack{\text{symptoms}\\ \text{develop, report}}}{\delta P_M} - \underset{\substack{\text{receive test}\\ \text{await results to isolate}}}{\omega_{P_M}} \tag{3}$$

$$\underset{\substack{\text{presymptomatic}\\ \text{eventually severe}}}{\dot{P_S}} = \underset{\text{become infectious}}{(1-p)q\sigma E} - \underset{\substack{\text{symptoms}\\ \text{develop, report}}}{\delta P_S} - \underset{\substack{\text{receive test}\\ \text{await results to isolate}}}{\omega_{P_S}} \tag{4}$$

$$\underset{\text{asymptomatic}}{\dot{A}} = \underset{\text{become infectious}}{p\sigma E} - \underset{\text{recover}}{\gamma_A A} - \underset{\substack{\text{receive test}\\ \text{await results to isolate}}}{\omega_A} \tag{5}$$

$$\underset{\text{mild symptoms}}{\dot{I_M}} = \underset{\substack{\text{symptoms}\\ \text{develop, report}}}{\delta P_M} - \underset{\text{recover}}{\gamma_M I_M} - \underset{\substack{\text{receive test}\\ \text{isolate immediately}}}{\omega_{I_M}} \tag{6}$$

$$\underset{\text{severe symptoms}}{\dot{I_S}} = \underset{\substack{\text{symptoms}\\ \text{develop, report}}}{\delta P_S} - \underset{\text{hospitalized}}{\eta I_S} - \underset{\substack{\text{receive test}\\ \text{isolate immediately}}}{\omega_{I_S}} \tag{7}$$

$$\underset{\substack{\text{awaiting results}\\ \text{presymptomatic, mild}}}{\dot{W_{P_M}}} = \underset{\substack{\text{receive test}\\ \text{await results to isolate}}}{\omega_{P_M}} - \underset{\substack{\text{recieve results}\\ \text{enter isolation}}}{\tau W_{P_M}} - \underset{\substack{\text{symptoms}\\ \text{before results}}}{\delta W_{P_M}} \tag{8}$$

$$\underset{\substack{\text{awaiting results}\\ \text{presymptomatic, severe}}}{\dot{W_{P_S}}} = \underset{\substack{\text{receive test}\\ \text{await results to isolate}}}{\omega_{P_S}} - \underset{\substack{\text{recieve results}\\ \text{enter isolation}}}{\tau W_{P_S}} - \underset{\substack{\text{symptoms}\\ \text{before results}}}{\delta W_{P_S}} \tag{9}$$

$$\underset{\substack{\text{awaiting results}\\ \text{asymptomatic}}}{\dot{W_A}} = \underset{\substack{\text{receive test}\\ \text{await results to isolate}}}{\omega_A} - \underset{\substack{\text{recieve results}\\ \text{enter isolation}}}{\tau W_A} - \underset{\substack{\text{recover}\\ \text{before results}}}{\gamma_A W_A} \tag{10}$$

$$\underset{\substack{\text{awaiting results}\\ \text{mild symptoms reported}}}{\dot{W_{I_M}}} = \underset{\substack{\text{symptoms}\\ \text{before results}}}{\delta W_{P_M}} - \underset{\substack{\text{recover}\\ \text{before results}}}{\gamma_M W_{I_M}} - \underset{\substack{\text{recieve results}\\ \text{enter isolation}}}{\tau W_{I_M}} \tag{11}$$

$$\underset{\substack{\text{awaiting results}\\ \text{severe symptoms reported}}}{\dot{W_{I_S}}} = \underset{\substack{\text{symptoms}\\ \text{before results}}}{\delta W_{P_S}} - \underset{\substack{\text{hospitalized}\\ \text{before results}}}{\eta W_{I_S}} - \underset{\substack{\text{recieve results}\\ \text{enter isolation}}}{\tau W_{I_S}} \tag{12}$$

$$\underset{\substack{\text{isolated}\\ \text{mild presymptomatic}}}{\dot{T_{P_M}}} = \underset{\substack{\text{recieve results}\\ \text{enter isolation}}}{\tau W_{P_M}} - \underset{\substack{\text{symptoms}\\ \text{after isolation}}}{\delta T_{P_M}} \tag{13}$$

$$\underset{\substack{\text{isolated}\\ \text{severe presymptomatic}}}{\dot{T_{P_S}}} = \underset{\substack{\text{recieve results}\\ \text{enter isolation}}}{\tau W_{P_S}} - \underset{\substack{\text{symptoms}\\ \text{after isolation}}}{\delta T_{P_S}} \tag{14}$$

$$\underset{\substack{\text{isolated}\\ \text{asymptomatic}}}{\dot{T_A}} = \underset{\substack{\text{recieve results}\\ \text{enter isolation}}}{\tau W_A} - \underset{\text{recover}}{\gamma_A T_A} \tag{15}$$

$$\underset{\substack{\text{isolated}\\ \text{mild symptomatic}}}{\dot{T_{I_M}}} = \underset{\substack{\text{receive test}\\ \text{isolate immediately}}}{\omega_{I_M}} + \underset{\substack{\text{recieve results}\\ \text{enter isolation}}}{\tau W_{I_M}} + \underset{\substack{\text{symptoms}\\ \text{after isolation}}}{\delta T_{P_M}} - \underset{\text{recover}}{\gamma_M T_{I_M}} \tag{16}$$

$$\underset{\substack{\text{positive, isolated}\\ \text{severe symptomatic}}}{\dot{T_{I_S}}} = \underset{\substack{\text{receive test}\\ \text{isolate immediately}}}{\omega_{I_S}} + \underset{\substack{\text{recieve results}\\ \text{enter isolation}}}{\tau W_{I_S}} + \underset{\substack{\text{symptoms}\\ \text{after isolation}}}{\delta T_{P_S}} - \underset{\text{hospitalized}}{\eta T_{I_S}} \tag{17}$$

$$\underset{\text{hospitalizated}}{\dot{H}} = \underset{\text{become hospitalized}}{\eta(I_S + W_{I_S} + T_{I_S})} - \underset{\text{death}}{\alpha H} - \underset{\text{recover}}{\gamma_S H} \tag{18}$$

$$\underset{\text{recovered}}{\dot{R}} = \underset{\text{mild symptom recovery}}{\gamma_M(I_M + W_{I_M} + T_{I_M})} + \underset{\text{asymptomatic recovery}}{\gamma_A(A + W_A + T_A)} + \underset{\text{hospitalized recovery}}{\gamma_S H} \tag{19}$$

$$\underset{\text{total population}}{\dot{N}} = -\underset{\text{death}}{\alpha H} \tag{20}$$

$$\tag{21}$$

with parameters defined in Table 1. When assuming that all individuals, including those with reported symptoms, wait until receiving a positive test result to isolate, Equations 11, 12, 16, and 17 become

$$\underset{\substack{\text{awaiting results}\\ \text{mild symptoms reported}}}{\dot{W_{I_M}}} = \underset{\substack{\text{receive test}\\ \text{await results to isolate}}}{\omega_{I_M}} + \underset{\substack{\text{symptoms}\\ \text{before results}}}{\delta W_{P_M}} - \underset{\substack{\text{recover}\\ \text{before results}}}{\gamma_M W_{I_M}} - \underset{\substack{\text{recieve results}\\ \text{enter isolation}}}{\tau W_{I_M}} \tag{22}$$

$$\underset{\substack{\text{awaiting results}\\ \text{severe symptoms reported}}}{\dot{W_{I_S}}} = \underset{\substack{\text{receive test}\\ \text{await results to isolate}}}{\omega_{I_S}} + \underset{\substack{\text{symptoms}\\ \text{before results}}}{\delta W_{P_S}} - \underset{\substack{\text{hospitalized}\\ \text{before results}}}{\eta W_{I_S}} - \underset{\substack{\text{recieve results}\\ \text{enter isolation}}}{\tau W_{I_S}} \tag{23}$$

$$\underset{\substack{\text{isolated}\\ \text{mild symptomatic}}}{\dot{T_{I_M}}} = \underset{\substack{\text{recieve results}\\ \text{enter isolation}}}{\tau W_{I_M}} + \underset{\substack{\text{symptoms}\\ \text{after isolation}}}{\delta T_{P_M}} - \underset{\text{recover}}{\gamma_M T_{I_M}} \tag{24}$$

$$\underset{\substack{\text{positive, isolated}\\ \text{severe symptomatic}}}{\dot{T_{I_S}}} = \underset{\substack{\text{recieve results}\\ \text{enter isolation}}}{\tau W_{I_S}} + \underset{\substack{\text{symptoms}\\ \text{after isolation}}}{\delta T_{P_S}} - \underset{\text{hospitalized}}{\eta T_{I_S}} \tag{25}$$

$$\tag{26}$$
