# Supplementary material for: Synergistic interventions to control COVID-19: Mass testing and isolation mitigates reliance on distancing
Source: PLoS Comput Biol. 2021 Oct 28;17(10):e1009518. doi: 10.1371/journal.pcbi.1009518 (PMC8553097; doi:10.1371/journal.pcbi.1009518)
Supplement: S1 Table — Initial conditions representing moderate levels of prior immunity at the time of intervention change. All W classes and T classes are represented by Wi and Ti, respectively. Increased levels of prior immunity were also considered in sensitivity analyses (denoted by *). (PDF) [file pcbi.1009518.s009.pdf]

S1 Table: Initial conditions. Initial conditions representing moderate levels of prior immunity at the time of intervention change. All W classes and T classes are represented by W_i and T_i, respectively. Increased levels of prior immunity were also considered in sensitivity analyses (denoted by *).

	Moderate Immunity	High Immunity*	Higher Immunity*
**Susceptible, S**	9380	8857	7857
**Exposed, E**	57	57	57
**Mild presymptomatic, P_M**	19	19	19
**Severe presymptomatic, P_S**	31	31	31
**Mild symptomatic, I_M**	1	1	1
**Severe symptomatic, I_S**	3	3	3
**Asymptomatic, A**	24	24	24
**Hospitalized, H**	0	0	0
**Recovered, R**	447	1000	2000
**Waiting for Results, W_i**	0	0	0
**Isolated, T_i**	0	0	0
**Dead, D**	8	8	8
